# Utopian or dystopian? The portrayal of the metaverse in popular news on social media

**DOI:** 10.1016/j.heliyon.2023.e14509

**Published:** 2023-03-22

**Authors:** Ruolan Deng, Jörg Matthes

**Affiliations:** Department of Communication, Faculty of Social Science, University of Vienna, Austria

**Keywords:** Metaverse, Media framing, Technology framing, Technology communication, Technology journalism, Hierarchy cluster analysis

## Abstract

The metaverse has sparked lots of interest worldwide as many giant tech companies are pursuing this futuristic idea. However, it has not been properly studied empirically by social science scholars yet. Considering the vital role played by media frames in affecting people's attitudes and behaviors towards the technology, this study examined the framing of the metaverse in popular news across Social Network Sites (SNSs) by cluster analyzing Entman's four operational frame elements. It identified five frames: economic prospect frame, unwanted future frame, consumer prospect frame, threatening future frame, and probable future frame. Overall, findings suggest a polarized framing of the metaverse on social media. While the majority of voices about the metaverse are optimistic, there is also a strong negative and dystopian perspective in more than one third of the SNS news. This positive or negative one-sided framing of the metaverse on SNSs may therefore fragmentize and polarize the audience, rather than informing in a balanced way. Implications for future research are discussed.

## 1 Introduction

After a hit of artificial intelligence, virtual reality (VR), mixed reality (MR), and all other cut-edge-technologies developed since the penetration of the internet in society in the 1990s, now a brand new concept – the metaverse, has gathered attention from the entire society worldwide as many giant tech companies are pursuing this futuristic idea. For example, on October 28, 2021, Mark Zuckerberg, CEO of Facebook, one of the largest social media platforms worldwide, announced that the corporate is rebranded as Meta [1]. The parent company is now called Meta platforms Inc. And before this formal announcement, the corporate has already invested billions to build a metaverse, transforming the online social media platform into a more realistic, immersive, and interactive home for remote work, study, and social interaction. This all-out movement to drive the metaverse of the leading tech company draws lots of attention from society. According to Google Trends,^1^ the interest of metaverse worldwide witnessed a breakout after Facebook's announcement and remained hot since then.

Other than Facebook, many huge tech companies like Microsoft, Amazon, Google, Nintendo, Epic Games, Nvidia, Apple, Disney, etc. are driving their metaverse future. And the metaverse is widely deployed by tech corporates as a buzzword for public relations purposes to draw in more investors [2]. A metaverse platform built by Epic Games in 2017, Fortnite, has witnessed a worldwide hit with assessing 350 million registered users in May 2020 [3]. Different from the immersive multi-player video games previously, in this game, the players can have a world of experience, like hanging out with friends to a concert or movie, buying land, creating your world guided by your rules, etc. In April 2020, more than 12 million players joined the Travis Scott Concert, an open and live-web experience on Fortnite where the musician and the players can interact with each other like in a real-life concert. The metaverse is seen as an alternative, especially when human contact is somehow limited physically during the pandemic. Another typical metaverse platform is Second Life, an online virtual community launched in 2003 where the players can create a unique avatar for themselves and have a “second life” online just like the game name suggested. The virtual representation of players, so-called avatars, can have all types of experiences within the virtual world, including shopping, trading, socializing, creating, studying, entertaining, etc. It is like an online extension of the real-life experience with a fully functioning economic and social ecosystem.

Companies from other industries, like entertainment, fashion, retailing, etc. are also transforming their current business system to the metaverse for promoting their brands, products, or services by emphasizing their initiative and innovative movement into the state-of-the-art technology. For many years, fashion brands and retailers were turning to the virtual dressing room to assist growing online customers to choose the fit, successively decreasing the return rates and increasing conversion rates. This was accelerated by the pandemic and lockdowns around the globe when customers were forced to buy online. The largest move was when Walmart acquired Zeekit, a virtual try-on tech startup in June 2021 [4]. And lots of fashion companies launched virtual products for customers’ virtual identities in virtual worlds. Some were designed specifically for avatars. A few can be worn for social media posting. Customers need to upload their photos and then match them with the virtual clothes automatically.

The term metaverse was originated from the novel book of Neal Stephenson [5] titled Snow Crash. In this novel, the metaverse is an escape of the main character from the unsatisfying real world. His customized avatar can wander around the digital world through certain devices. The virtual reality (VR) headsets that existed nowadays, like those from Meta Platforms Inc.‘s Oculus, have an identical function to the devices utilized by the main character in Snow Crash to access the metaverse. Although the definition of the metaverse has not reached a consensus in society yet at this early development stage, the term has been used currently to describe a parallel, perpetual, open, and connected real-time 3D virtual universe which interoperates more with the physical world. In a sense, it reaches the convergence of virtual and real-world [6]. The metaverse encapsulates many cutting-edge technologies like VR, augmented reality (AR), mixed reality (MR), artificial intelligence (AI), social networks, 5th generation mobile network (5G), blockchain, NFTs, cryptocurrency, etc. Nowadays, the internet consists of multiple unconnected and enclosed mini metaverse platforms, like Roblox, Meta (previously known as Facebook), Fortnite, Second Life, World Asset Exchange (crypto metaverse platform), so on so forth. But the ultimate goal of the metaverse is to create a connected seamless virtual universe that will bring a digital “big bang” to the prevailing cyberspace [7].

Technological development is of great social relevance in the world because it will change people's life significantly [8,9] or even bring a revolution to the whole society and humanity [10]. As argued by McLuhan in his technological determinism theory, technology determines the societal structure and cultures [11]. Although this theory was criticized for the absolute and exaggerated power of technological innovation, the influence of technology in society could not be denied in changing the interaction patterns. To some extent, the development of technology pushes the social revolution. But the impact of technology was not always beneficial. For instance, artificial intelligence (AI) has raised concerns over racial justice, gender equality, military weapons, AI taking over humans, etc. [12,13]. Moreover, people don't seem to be always well educated about the potential harm of the new technologies because the knowledge available for the general public on news media does not facilitate public discourse. Thus, the technological development in society requires a communications study approach to guarantee that it will be adopted properly to accelerate social development but at the same time, avoid the potential harm to humanity and society.

Although this term, metaverse, came up some time ago and has recently attracted considerable international attention, it has not been thoroughly studied empirically by social science scholars. The prevailing studies were concentrating on explaining the metaverse and also the social changes it has entailed [[14], [15], [16]], exploring the new possibilities of the metaverse [[17], [18], [19]], developing a framework for future research [7], and suggesting a remedy or solution for the potential problems [20]. However, hardly any studies empirically examined the metaverse from the perspective of users which was the core determinant of emerging technology's development and final deployment.

Since the common notion predict that younger generations are the main force in driving new technology adoption process [21,22], this paper analyzed how the highly engaged news on social media platforms are framing the metaverse because SNSs are the dominant source for youngsters to receive information rather than the traditional news brands [23]. By cluster-analysing Entman's four operational framing elements, this paper aimed to search out how the metaverse is framed in popular news content on social media and what is influencing the general public perceptions of the metaverse. This research intended to fill the knowledge gap of how journalists build their hegemony of the metaverse and shape the public discussion. And practically, the technology communication bodies, like giant tech corporations, policymakers, researchers, social activists, etc., can devise campaigns strategically and responsively to the existing metaverse frames in popular news on social media.

## 2 Literature review

### 2.1 Social construction of technology

The social construction of technology (SCOT) is an agency-centred approach to clarify the developmental process of technology, just like the emergence of the latest technology. Besides the technical requirement, a brand new technology's success is additionally dependent on relevant social actors. Gash and Orlikowski [24, 25] identified seven relevant social groups in organizations based on their stake holdings, including managers, technologists, vendors, consultants, customers, users, and government regulators. Humphreys [26] organized relevant actors into producers, advocates, users, and bystanders according to their interactions with the technology. Illuminated with the instance of bicycle, SCOT assumes that different social groups can assign different meanings to the identical technology, so-called “interpretive flexibility” [27,28]. There is no commonly agreed definition of technology across social groups at the start of its lifecycle. People negotiate along the way through interacting with other groups or with technology. Then they react to the technology in their unique way, which either pushes or disrupts the success of the new technology and affects the planning of the technological artefacts.

Powerful social groups, like CEOs, industrialists, policymakers, journalists, etc. can interpret technologies based on their values and beliefs, formulating hegemony over the definition of the latest technology. As claimed by Pinch and Bijker [29], the crucial requirement for identifying a social group is that “all members of a certain social group share the same set of meanings, attached to a specific artifact” (p. 30). In the development process of recent technology, journalists should be considered jointly as the important relevant social group for his or her common role and goal of disseminating important information to the general public and facilitating public discourse [30]. Zelizer [31] argued that rather than treating journalists as a profession, they act more like an interpretive community. They formulated and spread meaning to a specific event or issue.

Based on Zaller's [32] definition of elites, journalists are considered a group among them who devote themselves to political issues and public affairs and are relied on by the public for information (p. 6). Brennen and his colleagues [33] also found that journalists expressed their values and opinions in many strategic ways to shape audiences' expectations and interpretations of an issue, like the selection of sources, the arrangement of the article, etc. Journalists don't seem to be just information providers; they ought to also take social responsibility and facilitate public discourse [30]. A healthy public discourse of emerging technology is vital because technology significantly affects the way people live and work [8] and even the evolutional process of the entire society and humanity [10].

### 2.2 Framing theory

Conceived first by Goffman [34] in his book, frames are inevitable tools people utilize to perceive and comprehend everything around them and this is often determined by the entire culture and society. People refer to their primary framework consciously or sometimes unconsciously to speed the interpretation process of reality every day. He proposed to apply frame analysis as a research method in ethnographic studies to understand how people make sense of issues and events in the world. The idea of frame analysis and framing theory played a very important role in media and communication research then, especially in analyzing frames in news media. Framing theory presumes that when viewing from different frames, a selected issue or object has various meanings and implications [35]. Therefore, people will assign different values and respond to the same situation diversely.

In content analysis studies, framing is defined as “the process by which a communication source, such as a news organization, defines and constructs a political issue or public controversy” [36], (p. 221). The powerful communication parties exploit frames to define reality for people with less influential communication resources. During the framing process, certain aspects of the issue or object are selected or highlighted while the other aspects were ignored purposely or unintentionally [37]. Similar to agenda setting [38], framing demonstrates media power in affecting and even determining people's perceptions and interpretations of the reality in the world, especially when they do not have direct experience with it. The discrepancy lies in the consequence brought by these two processes. Agenda setting intends to impact audiences' perception of what issue is more important and salient among all events happening around the world. While framing acts on a deeper cognition of a specific issue or object, like the nature of it, values assigned to that, memories about it, and at the end, swaying their attitudes and responses to it [39].

In communication science, the most commonly-used and influential definition of framing is from Entman [40] because it offers operational framing elements compared to previously vague and abstract definitions, namely problem definition, causal diagnosis, moral evaluation, and treatment recommendation. A problem definition recognizes the consequences of the main actor's action in an exceedingly commonly accepted sense; causal diagnosis pinpoints the reasons for that problem; moral evaluation refers to the judgement towards the issue; treatment recommendation means the future solution proposed for the matter. He argued that to frame is to select and accentuate certain aspects of an issue while communicating whether on purpose or not. Therefore, audiences were merely shown a segment of the entire reality which can change their perceptions and understanding of the issue (noted as schema) [40]. This operational framing prototype was originally designed for analyzing political issues.

### 2.3 Technology framing analysis

Scholars acknowledged the merit of Entman's framing paradigm in frame analysis of emerging technology as well [13,41,42]. In Chuan, Tsai, and Chou's framing analysis of artificial intelligence in five American newspapers from 2009 to 2018, they coded topics, evaluation, and risks and benefits as separate variables [41]. The topics included eight key codes extracted from previous research: education, entertainment, science fiction, threat, ethics, politics, business & economy, technology development & application. The evaluation was categorised into positive valence, negative valence, and mixed valence. And they specified the benefits of AI into the economy, well-being, and reduced bias and risks into shortcomings, ethical concerns, Pandora's box, run-away-train, misuse, privacy concern, embedded bias, and loss of jobs.

Instead of observing patterns of each variable separately, Köstler and Ossewaarde [13] extract unobservable holistic frames based on these four theoretical and operational framing elements. Following the guidance of Entman's [40] original theory, they categorized problem definition into risks & challenges, benefits & chances; derived three broad groupings of causal diagnosis from past literature – external influence (e.g. foreign competition), national qualities (e.g. scientific expertise), and technological process (e.g. autonomous development); classified moral evaluation into desirable, threatening, and probable; identified four suggested solutions – the requirement of further investment, the request to cooperation, the demand for public debate, and also the call of regulations, standards, and guidelines. Guided by this comprehensive framework, they conducted a comparative content analysis of government documents and newspaper articles. They revealed that German newspapers adapted the AI future frame in the government document to make it more neutral.

To improve the reliability and validity of framing analysis, Matthes and Kohring [42] suggested a cluster analysis method of Entman's four operational frame elements to extract the frames in news articles. They considered frames the certain patterns of how these elements grouped systematically. Only the frame elements were coded into dummy variables for every article. Exemplified with biotechnology, they grouped problem definition into nine topics (genetic identity, regulation, public opinion, moral, cloning, economics, research, agriculture, biomedicine) and four actors (politics, business, media/public opinion, science), causal diagnosis into three actors (politics, business, science) for benefits and risks attribution each, moral evaluation into five benefits (consumer, legal, research, economic, health) and two risks (health, moral), treatment recommendation into negative (i.e. to stop) and positive judgement (i.e. not to stop). And hierarchical cluster analysis was applied to determine the number of frames based on the principle of commonality within the cluster and the disparity across the clusters. Finally, they recognized three frames from the cluster analysis with New York Times news coverages of biotechnology in period 1992 to 1996 – economic prospect frame, genetic identity frame, and research benefit frame and six frames from the cluster analysis with news coverages in period 1997 to 2001 – economic prospect frame, biomedical prospect frame, biomedical research frame, research benefit frame, genetic identity frame, Agri-Food: pros & cons frame.

In the whole lifecycle of technology, frames that people refer to have a robust association with their final choices on accepting or refusing this technology [43]. In their study of discontinuance of volitional information system (IS), they identified two frames – the hedonic frame and the gain frame. These two frames explained the disparity of users' interpretation and evaluation of IS. When their dominant frame of technology was the hedonic frame, they cared more about the entertainment value brought by IS. In contrast, its instrumental benefit was the central concern if the gain frame was the primary frame in their mind. The fulfillment of their primary goal with the technology ends up in the choice of IS usage. This cognitive process could be modified by other communication sources' framing of the emerging technology, such as journalism, government documents, and technology companies' release, thus shaping their attitudes and intended actions towards it [33,41,[44], [45], [46]]. Deriving from framing theory, scholars also conducted experimental studies to check media framing's influence on participants' attitudes and responses to technology [47]. Participants shown to “AI as social progress frame” message were more likely to support artificial intelligence (AI) than participants shown to “AI as Pandora's box frame” message. The result proved that media framing could shape audiences' schema through selection and accentuation of certain aspects of the technology. This schema change consequently models their opinion formation and reactions. Therefore, examining the metaverse frames is helpful to understand what media frames are affecting the general public perceptions and attitudes towards this emerging technology. Against this background, this study takes an exploratory approach analyzing metaverse frames in popular news across SNSs.

## 3 Methodology

### 3.1 Methods

Frame analysis is usually utilized by scholars to explore the power of the government, social elites, and news media on influencing the public's cognitive sense-making of the perceived reality of a selected issue or event [37,48,49]. It also provides a solid background to study the new technology and its applications theoretically, methodologically, and critically [13,[50], [51], [52]] when the purpose is to elucidate the underlying mechanism of people's attitude and behaviour towards it. Therefore, this paper analyzed the entire metaverse frame in each news article following the operational guidance of Entman's framing paradigm [40]. The unit of frame analysis is the entire news article like what other studies have done before [13,42].

This paper adopted the improved methodology for the content analysis of media frames proposed by Matthes and Kohring [42] which treated media frames as clusters of four framing elements to extract overall frames of the metaverse in highly engaging news articles on social media. Compared to the holistic frame analysis method commonly used over the past decades, this framing approach could improve the results' reliability and validity [42] because of two reasons. First, instead of coding holistic frames, content analysis of four concrete framing elements could increase reliability as the extent of manifestation of a variable is proportional to the level of reliability [53]. Second, clustering the frame elements could eliminate coders' expectations in the inductive extraction of frames. Therefore, the ultimate results will be less littered with coders' subjective bias compared to drawing out abstract frames directly from the article. Another advantage of this method is that new emerging frames can be easily detected without demanding researchers’ massive cognitive resources to spot nuances of various metaverse frames.

### 3.2 Sampling

The samples were collected on the website BuzzSumo, a cloud-based platform tracking and archiving the web content performance regarding its social sharing and engagement across SNSs, like Facebook, Twitter, Pinterest, and Reddit. These four social media platforms are among the most popular SNSs in the world. In 2021, there were estimate 2.91 billion monthly active users (MAU) on Facebook [54], 217 million MAU on Twitter [55], 431 million MAU on Pinterest [56], and 1.7 billion visits on Reddit [57]. When searching a selected topic or keyword, all relevant content is listed according to the engagement rate measured by viewing, sharing, and commenting. This user-friendly and free website is widely utilized by marketers and campaigners as social media analytics and curation tool. It tracks the social likes and shares of relevant web content in any specific field. For more information on the technical problems of this social engagement tracking website, the developer's guidance website offers further guidance: https://help.buzzsumo.com/en/

This platform has also been widely employed by scholars to gather most shared news on social media regarding a particular topic (see e.g., [[58], [59], [60], [61]]). For example, Biancovilli and her colleagues [58] conducted content analysis with the most shared news stories on social media after putting in keyword “breast cancer” on BuzzSumo. Waszak and his colleagues [61] collected 80 samples from keyword search on BuzzSumo to detect the presence of medical fake news on social media. Obiała et al. [59] identified 30 most shared news articles about COVID-19 prevention and classified their accuracy.

The keyword (metaverse) was placed on the BuzzSumo keyword search, setting the time frame to the past year, geography to worldwide, language to English, and type to journalist's content only. The top 100 news content automatically ranked by the engagement criteria were selected for further screening. We determined the sample sizes based on the following reasons: first, those top engaging news stories had greater visibility and reach on studied social media platforms. Thus, they were more relevant to answer our research question regarding the influential media frames of the metaverse on social media. Second, this cut would make it manageable for frame analysis. The previous quantitative content analysis had a similar sample size around 100 most shared news [[58], [59], [60], [61]].

In our study, we made the keyword search three times over two months. The first search yielded 99 news on Jan 1, 2022. The same process was then performed on Jan 15, 2022, and February 15, 2022. The news links that overlapped were removed. Only the news with the majority attention on the metaverse topic went to final manual frame elements coding. The news could be in text, video, or a combination of formats. Finally, 202 pieces of news were gathered for analysis; see Appendix A for further information. All these news contents received over 6 thousand shares across those social media networks. The majority of the sample news were from American or British news organizations. Six news were from sources in other regions, including Canada, Netherlands, Portugal, India, Saudi Arab, and Iceland.

### 3.3 The procedure of cluster analysis

The fundamental idea of this method was that in a unit of text, frame elements grouped systematically and several other units of text shared a particular pattern. These emerging patterns identified in a sample were referred to as frames [42]. Instead of analyzing the abstract holistic frame, this paper coded four operational frame elements defined by Entman [40] in a given text: problem definition, causal diagnosis, moral evaluation, and treatment recommendation. And every frame element contained multiple analytical dummy variables. Problem definition here identifies the consequences of the metaverse in a good or bad way. Causal diagnosis interprets the actors attributed to the problem. Moral evaluation evaluates the metaverse. And treatment recommendation suggests remedies to the problem.

Following the theoretical guidance of Entman's framing definition, the codebook was tailored to the context of the metaverse from earlier content analysis of other technologies (e.g., [13,41,42]). The codes were created strictly following the principle that they should be mutually exclusive and independent and collectively exhaustive. Problem definition consists of two benefits (consumer and economics), three risks (ethics, wellbeing, and out of control), and one challenge. The causal diagnosis includes two attributions (business actors and technology concept itself) for benefits, risks, and challenge each. Thus, there are six categories for causal diagnosis. Moral evaluation treats the metaverse as desirable, threatening, unwanted, and probable. And treatment recommendation calls for four types of actions, namely investment, regulation, stop, and debate. Each code under the four frame elements stands as one binary analytical dummy variable. Appendix B contains the detailed codebook instructions.

Two coders were recruited for the manual frame elements analysis. Before coding the actual sample, they were trained for the instruction codebook and then coded 30 random news articles about the metaverse from internet search independently. Inter-coder reliability was estimated using Krippendorff's alpha [62]. The majority of variables generated a relatively high inter-coder reliability (α > 0.80). Five dummy variables' inter-coder reliability was acceptable as their alpha value almost reached 0.80. Only one variable (causal diagnosis: Risk-business actors) yielded a relatively low inter-coder reliability (α = 0.68). There was only one case where the two coders disagreed on this variable. But the others were nearly all coded as zero. Therefore, a single disagreement could lower the alpha value. If considering the simple agreement level, the inter-coder reliability for this variable should be acceptable.

After manually coding frame elements, a hierarchical cluster analysis (Ward's method) was conducted using SPSS version 25 to explore relatively homogenous groups of cases based on certain attributes. The Ward method [63] is an appropriate and accurate tool for identifying clusters [64,65]. The distance and similarity were measured with Euclidean distance (the square root of the sum of the squared differences between values for the items). The algorithm begins with a single case in one cluster and gradually combines cases into the same cluster depending on the proximity of selected characteristics until all cases were included in one cluster.

## 4 Results

The hierarchical cluster analysis with Ward's method analyzed the heterogeneity and proximity among all cases. There were 16 clusters at the very beginning. It agglomerated into 10 clusters at rescaled distance 2. All cases were divided into seven clusters at distance 3. It merged into five clusters at distance 4. There were three clusters after distance 8. At distance 10, three clusters consolidated into two clusters. All cases were included in a single large cluster at distance value 25. Since this study is among the first attempts to explore the metaverse frames, it aims to extract as many exhaustive frames as possible under the premise of heterogeneity and interpretability. The distance across groups became relatively more heterogeneous between five clusters and three clusters (Euclidean distance is from 4 to 8). Therefore, we concluded five clusters for all these cases. We also compared the interpretability with seven clusters and 10 clusters solution. The five clusters solution was superior to these solutions in terms of clarity and interpretability.

We summarized the mean values of each dummy variable in these five clusters in Table 1 to illustrate the difference of each cluster along with each frame element characteristic. The mean values help to understand the most important characteristics of each frame as well as the salient difference across frames. For example, the most noticeable features of the first frame were the economical benefits, attribution of this benefit to the technology itself, evaluating the metaverse as a desirable future, and the call for more investment into it. The third frame had the same moral evaluation and treatment recommendation. But the striking differences were that the third frame focused on the benefit brought to consumers and attributed this benefit to the technology itself and business actors almost equally.

**Table 1:** Mean values and standard deviation for five identified frames.

Variable	Economic prospect (24.26%)	Unwanted future (20.79%)	Consumer prospect (20.30%)	Threatening future (18.32%)	Probable future (16.34%)
Benefit: consumer^1^	0.00 (0.00)	0.00 (0.00)	0.90 (0.30)	0.00 (0.00)	0.70 (0.47)
Benefit: economics^1^	1.00 (0.00)	0.00 (0.00)	0.56 (0.50)	0.00 (0.00)	0.61 (0.50)
Risk: ethics^1^	0.00 (0.00)	0.90 (0.30)	0.00 (0.00)	0.89 (0.32)	0.61 (0.50)
Risk: wellbeing^1^	0.00 (0.00)	0.29 (0.46)	0.00 (0.00)	0.70 (0.46)	0.15 (0.36)
Risk: out of control^1^	0.00 (0.00)	0.00 (0.00)	0.00 (0.00)	0.30 (0.46)	0.24 (0.44)
Challenge^1^	0.00 (0.00)	0.74 (0.45)	0.02 (0.16)	0.05 (0.23)	0.42 (0.50)
Benefit: business^2^	0.00 (0.00)	0.00 (0.00)	0.66 (0.48)	0.03 (0.16)	0.70 (0.47)
Benefit: technology^2^	1.00 (0.00)	0.00 (0.00)	0.59 (0.50)	0.00 (0.00)	0.30 (0.47)
Risk: business^2^	0.00 (0.00)	1.00 (0.00)	0.00 (0.00)	0.78 (0.42)	0.64 (0.49)
Risk technology^2^	0.00 (0.00)	0.00 (0.00)	0.00 (0.00)	0.27 (0.45)	0.18 (0.39)
Challenge: technology^2^	0.00 (0.00)	0.00 (0.00)	0.02 (0.16)	0.03 (0.16)	0.27 (0.45)
Challenge: business^2^	0.00 (0.00)	0.74 (0.45)	0.00 (0.00)	0.03 (0.16)	0.18 (0.39)
Desirable^3^	1.00 (0.00)	0.00 (0.00)	1.00 (0.00)	0.00 (0.00)	0.00 (0.00)
Unwanted^3^	0.00 (0.00)	1.00 (0.00)	0.00 (0.00)	0.08 (0.28)	0.00 (0.00)
Threatening^3^	0.00 (0.00)	0.00 (0.00)	0.00 (0.00)	0.92 (0.28)	0.00 (0.00)
Probable^3^	0.00 (0.00)	0.00 (0.00)	0.00 (0.00)	0.00 (0.00)	1.00 (0.00)
Investment^4^	1.00 (1.00)	0.00 (0.00)	1.00 (1.00)	0.00 (0.00)	0.00 (0.00)
Regulation^4^	0.00 (0.00)	0.00 (0.00)	0.00 (0.00)	0.51 (0.51)	0.00 (0.00)
Debate^4^	0.00 (0.00)	0.00 (0.00)	0.00 (0.00)	0.00 (0.00)	1.00 (0.00)
Stop^4^	0.00 (0.00)	1.00 (0.00)	0.00 (0.00)	0.49 (0.51)	0.00 (0.00)

1 – codes under problem definition element 2 – codes under causal diagnosis.

3 – codes under moral evaluation 4 – codes under treatment recommendation.

The first frame, economic prospect, was the biggest cluster (24.26%) in these popular news stories on social media. This frame concentrated on the economic prospects of the metaverse. Within this frame, the metaverse represented enormous economic opportunities for all businesses worldwide. A massive market appeared because of the metaverse. The metaverse itself took the credit for the economic benefits. Morally the metaverse was deemed a desirable futuristic idea. And this frame called for more investments into the metaverse.

Unlike the economic prospect, the second frame, unwanted future (20.79%), drew extensive attention to the ethical risks and challenges of the metaverse. The business actors were identified as the responsible actors. No matter the ethical issues or the challenges problem, business actors who build the metaverse were seen as the sole contributors. The most prominent traits of the unwanted future frame were the moral evaluation and treatment recommendation element. Compared to other frames, the unwanted future frame scored the highest on the unwanted for moral evaluation dimension and stop for treatment recommendation dimension.

In terms of the moral evaluation and treatment recommendation, the third frame, consumer prospect (20.30%), was similar to the first frame, economic prospect. They were all desirable and deserved more investments. However, it differed from the economic prospect in the problem definition and causal diagnosis dimension. Within this frame, the majority centered on the benefits of the metaverse for consumers. Some of them still mentioned the economic benefits. Business actors and the metaverse itself both took credit for these benefits. But business actors outweighed the metaverse itself as the contributor by a slight margin.

The next frame was the threatening future frame (18.32%). This frame highlighted the risks of the metaverse. But the score for the problem of challenges was far lower than the unwanted future frame. The majority of the news within this frame portrayed business actors as the agent for the problem. Around a quarter of them also depicted the metaverse itself as the cause of the problem. The distinguishing characteristics of this frame were the moral evaluation dimension and the treatment recommendation dimension. Nearly all of them evaluated the metaverse as a threatening future. Half of them suggested properly regulating the problems. The other half recommended stopping the metaverse immediately.

The final frame was named the probable future frame (16.34%) because the metaverse here was regarded as probable. It may or may not come to fruition in the future. These news stories cared about the desirable sides and the unfavorable sides of the metaverse equally. The benefits, risks, and challenges were all disclosed to audiences. For every kind of problem, both business actors and the metaverse itself were blamed but the business actors were held more accountable than the metaverse. Within this frame, the treatment recommendation was not given directly by journalists. But the news implicitly requested a thorough debate about the metaverse since it displayed more balanced viewpoints without inclination to any side.

## 5 Discussion

### 5.1 Metaverse frames

The findings present the existence of various views towards the metaverse, including the extreme optimism (44.56%) and pessimism (39.11%) to the metaverse, and also the paradoxical point of view (16.34). In these popular news stories of the metaverse on social media, the metaverse was framed as either utopian, dystopian, or duality at the beginning of the metaverse discussion in news media. Similarly, in the history of the academic sphere, some scholars [66,67] perceive the technology with fully complementary remarks like freedom, assimilation, competence, engaging, etc. Whereas, some [68,69] view the technology strictly in critical terms like enslavement, isolation, incompetence, disengaging, etc. And a couple of them [[70], [71], [72]] evolved into a paradoxical perspective with a more balanced understanding taking both costs and benefits of the technology into consideration.

The leading role of economic prospect frame (24.26%) among these five frames further substantiates the earlier argument that “the constant imagination and pursuit of distant futures has been repeatedly identified as a central dynamic of capitalism” [73], (p. 1956). Even though the metaverse is still a futuristic idea that may or may not come to fruition, more and more businesses are seizing the financial prospects to construct a distant internet future. Metaverse is just a stylized term propagated by large corporations to describe the imaginary possibilities of a future internet world in the face of uncertainty. The media's disproportionate focus on tech behemoths' pursuit of a distant metaverse future exemplifies the existing communication power's disparity and inequality.

More news (44.56%) was optimistic towards the metaverse, focusing on its benefits to consumers and economic possibilities. This finding corresponds with a previous long-term technological frame study [45] which indicates that at the initial stage, the overall tone towards the emerging technology was enthusiastic but it became more balanced and critical in the following years. When technological innovation is first introduced to the public, the public tends to associate that newness with societal progress and advancement [74], (p. 404). Moreover, before the innovation is implemented in real life, there is no way to know what the harms it will bring. Therefore, at this early stage, the predominant voice towards the metaverse is optimistic in news media.

Although not exceeding the number of optimistic news, a substantial portion of news also framed the metaverse as dystopian (39.11%). They described the possible metaverse future as unpleasant and dehumanizing. These news stories were fully one-sided and emphasized either the existing problems or the future threats. The portrayal of the metaverse as the dystopian in news media deserves attention because this could translate to public overreactions to its potential harm, which will then slow or halt the adoption of the technology [75]. Exaggeration of the risks or flaws of the metaverse is a dangerous indicator since it has the potential to turn the public against the metaverse.

The findings demonstrate that a large number of popular news articles (83.66%) related to the metaverse on social media are divided into opposite stands. Rather than fostering a healthy public debate over the metaverse, the majority of popular news on social media is dominated by strong opinions and arguments on one side of the story. In comparison to the loud polemics of either utopian or dystopian metaverse, the volume of a deeper and more extensive analysis of the metaverse was relatively rare. This is consistent with the argument that social media contributes to the increased polarization of viewpoints because of homogenous social networks, personalized filterer algorithms, social feedback and reinforcement, and other factors [[76], [77], [78]]. Hong and Kim [76] designed a study and proved the association between the U.S. House of Representatives' ideological position and the size of their Twitter readership. Their results revealed that the more polarized the politicians’ views are, the more followers they get on Twitter. In light of the findings of this study, it appears that on these fragmented social media platforms, the content (whether from the government or the news media) with an extreme viewpoint regarding a certain issue will receive more attention and popularity from audiences.

### 5.2 Implications

The finding highlights the importance of an open debate on the metaverse in the co-creation of a possible internet revolution. In other words, public interests should take precedence over corporate interests concerning technological innovations to prevent capitalism's domination in society. New technological innovations should aim for doing the public good in the end instead of bringing financial benefits to private companies.

Considering the large portion of popular news against the metaverse on social media, the practitioners will put far more effort to respond to it so that the adoption process of the metaverse could be smoother. They should design strategic communication campaigns to address public concerns about the metaverse so that these dystopian metaverse frames in news media will not lead to a backlash against the metaverse.

For content producers, the important implication is that they have to express a strong and even extreme argument rather than a moderate and weak position to gain a wider spread on social media. People are more likely to share and engage with news that has strong opinions on social media since this study finds that the majority of the popular news on social media portrayed the metaverse as either utopian or dystopian. The polarized content seems to grasp more eyeballs on social media. On the other hand, it warns that policymakers should be cautious to collect and translate citizen views from social media where the popularized views float everywhere.

Additionally, an alternative approach is needed to allow for more exhaustive narratives surrounding the metaverse. Social media is not such a space for its affordance of fragmented and polarized ideology. A more inclusive and tolerant open space should be created for dialogues and engagements so that new stories and perspectives can emerge from the discussion. To prevent chaotic partisan squabbling, it would be preferable to have a panel of moderators to regulate the discussion and direct it to a rational, reasonable, dynamic, and intelligent discourse.

Furthermore, this finding points out the urgency of more effort into investigations over the metaverse on both sides. The research centers, journalists, and other social activists should delve deeper into the metaverse to inspect its promises and potential harms and consequences. With additional knowledge sources besides those renowned tech leaders, the metaverse news could formulate a fairer dialogue. And the general public could get access to more diversified information to construct a healthy public discourse which is necessary for critical assessment and justified choice of the metaverse, given the influence of news on people's attitudes towards emerging technology.

Methodologically, this study implies the robustness and resilience of the alternative method proposed by Matthes and Kohring [42] in a different context – media framing of the metaverse. It suggests that with a solid theoretical basis offering operational frame elements (in this case, Entman's four framing functions), cluster analysis could easily identify all interpretable frames independent of the media context. There are certain patterns of how these frame elements combine systematically to form a media frame. Compared to manual holistic media frame extraction, this method improves the validity and reliability of frame analysis studies. The different researchers will get the same clusters across samples when they repeat the study. Moreover, the codebook developed at this stage could lay the foundation for future metaverse framing studies. The codebook will probably become larger and more complex than the current one as the metaverse develops and more stories about the metaverse are published.

### 5.3 Limitations

Though the approach seemed appropriate to solve the research question, the study has certain limitations. First, collecting samples from BuzzSumo has a liability problem with the algorithm used to determine the engagement rate of new articles. Although many studies have recently employed this method for their social media research [58,61], the nontransparent ranking rules may still raise criticism. And we don't know if all relevant articles are achieved in its database. Related to that, there may be strong country or cultural differences that we were unable to analyze. Future research should therefore employ systematic sampling strategies, leading to larger and more valid samples.

Additionally, this study only illuminates the metaverse frame in the early phases. And the codebook is only designed for the news during the hit discussion after Facebook's rebranding. How the frames evolve over time needs further investigation. New codes for each frame element and new patterns of the frame elements may emerge in the future as the stories over the metaverse shift their focus. Therefore, future research could examine the long-term changes and trends of the metaverse frames, which could shed light on the relationship between modern technology and the public through social media. Finally and related to that, more research is needed on how metaverse frames on SNSs impact audience members, ideally taking a diachronic [48] and cross-cultural perspective.

## 6 Conclusion

Since the metaverse is a new concept to the general public and a new topic in the academic sphere, this study intends to fill in this knowledge gap on media frames of the metaverse. The sample consists of popular pieces of news on social media because they are assumed to have a greater influence on the young generation. Using cluster analysis, this study discovered five metaverse frames in the popular news on social media: economic prospect frame, unwanted future frame, consumer prospect frame, threatening future, and probable future frame. The majority of these news stories were one-sided: either picturing the metaverse as utopian or dystopian. The two extreme stands had nearly the same volume of news on social media. The findings highlight the importance of additional research and investigations into this emerging technology. As a result, the news media could have access to a broader range of sources rather than being limited to those tech behemoths. The public will be fed with more balanced and neutral information. This, in turn, leads to an open and healthy debate about the metaverse which is essential to critical assessment and justified choice of the metaverse.

## Author contribution statement

LineNoBookmarkStart:ID:78 = Name:Line_manuscript_77].

Ruolan Deng: Conceived and designed the study; performed the study; analyzed and interpreted the data; wrote the paper.

Jörg Matthes: Conceived and designed the study; interpreted the data; wrote the paper.

## Funding statement

This research did not receive any specific grant from funding agencies in the public, commercial, or not-for-profit sectors.

## Data availability statement

Data will be made available on request.

## Conflict of interest

The authors declare that they have no conflict of interest.

## Figures and Tables

**Table:** 

No.	Link	Sources	Source Country	Engagement/K
1	https://www.theverge.com/22588022/mark-zuckerberg-facebook-ceo-metaverse-interview	The Verge	America	93.1
2	https://www.businessinsider.com/playstation-inventor-metaverse-pointless-2022-1	Insider	America	83.9
3	https://www.businessinsider.com/mark-zuckerberg-the-metaverse-golden-goose-2022-1	Insider	America	77.6
4	https://www.theverge.com/2021/10/19/22735612/facebook-change-company-name-metaverse	The Verge	America	57.6
5	https://www.businessinsider.com/metaverse-hype-communist-propaganda-evernote-founder-phil-libin-soviet-union-2022-1	Insider	America	46.7
6	https://www.cnbc.com/2022/01/18/microsoft-to-buy-activision.html	CNBC	America	45.2
7	https://www.coindesk.com/business/2021/10/20/metaverse-backer-animoca-brands-raises-65m-at-22b-valuation/	CoinDesk	America	44.9
8	https://nymag.com/intelligencer/2021/11/why-facebooks-metaverse-is-dead-on-arrival.html	New York Magazine	America	44.3
9	https://futurism.com/chatbot-abuse	Futurism	America	36.5
10	https://www.coindesk.com/layer2/2022/01/08/the-metaverse-needs-a-constitution/	CoinDesk	America	35.3
11	https://www.theguardian.com/lifeandstyle/2021/oct/29/metaverse-zuckerberg-mars-billionaires-escape-world	The Guardian	Britain	34.2
12	https://www.businessinsider.com/former-google-ceo-expresses-concerns-about-facebook-metaverse-2021-10	Insider	America	33.6
13	https://futurism.com/the-byte/650000-nft-yacht-metaverse	Futurism	America	29.3
14	https://mashable.com/article/apple-metaverse-facebook-ar-vr-headset	CoinDesk	America	29.3
15	https://www.coindesk.com/business/2021/11/04/enjin-forms-100m-fund-to-support-metaverse-projects/	CoinDesk	America	27
16	https://www.coindesk.com/layer2/2022/01/05/facebook-walmart-and-how-companies-shouldnt-set-up-in-the-metaverse/	CoinDesk	America	26.6
17	https://www.coindesk.com/tech/2022/01/09/binanceus-is-building-an-office-in-the-solana-metaverse/	CoinDesk	America	26.5
18	https://www.coindesk.com/business/2021/12/13/sk-square-to-demand-metaverse-presence-for-portfolio-companies-report/	CoinDesk	America	25.9
19	https://www.reuters.com/technology/microsoft-buy-activision-blizzard-deal-687-billion-2022-01-18/	Reuters	Britain	24.9
20	https://www.businessinsider.com/bill-gates-virtual-meetings-metaverse-remote-work-virtual-reality-2021-12	Insider	America	24.3
21	https://www.cnbc.com/2022/01/12/investors-are-paying-millions-for-virtual-land-in-the-metaverse.html	CNBC	America	24.2
22	https://www.businessinsider.com/facebook-meta-metaverse-splinter-reality-more-2021-11	Insider	America	24.1
23	https://www.businessinsider.com/metaverse-silicon-valley-tech-data-collection-regulation-laws-need-updating-2021-12	Insider	America	24
24	https://www.breitbart.com/tech/2022/01/05/egg-on-zucks-face-facebooks-metaverse-concerts-all-flopped/	Breitbart	America	23.1
25	https://www.coindesk.com/policy/2021/11/19/why-barbados-metaverse-embassy-matters/	CoinDesk	America	22.9
26	https://www.cnbc.com/2021/12/24/baidu-says-it-could-be-6-years-before-it-can-fully-deliver-its-metaverse.html	CNBC	America	22.9
27	https://www.coindesk.com/business/2021/11/02/metaverse-startup-the-sandbox-closes-93m-series-b-led-by-softbank/	CoinDesk	America	22.9
28	https://www.theguardian.com/games/2022/jan/25/ive-seen-the-metaverse-and-i-dont-want-it	The Guardian	Britain	22.7
29	https://www.coindesk.com/markets/2022/01/17/land-prices-surge-on-cardano-metaverse-project-pavia/	CoinDesk	America	22.1
30	https://www.cnbc.com/2022/01/01/meta-apple-google-microsoft-gear-up-for-big-augmented-reality-year.html	CNBC	America	21.8
31	https://www.businessinsider.com/metaverse-facebook-china-big-tech-alibaba-tencent-2021-10	Insider	America	21.3
32	https://www.cnbc.com/2021/12/09/bill-gates-metaverse-will-host-most-virtual-meetings-in-a-few-years.html	CNBC	America	20.9
33	https://www.zdnet.com/article/zuckerberg-unveils-facebooks-horizon-metaverse/	ZDNet	America	20.4
34	https://futurism.com/elon-musk-metaverse-sucks-neuralink-better	Futurism	America	20.2
35	https://www.theatlantic.com/technology/archive/2021/10/facebook-metaverse-name-change/620449/	The Atlantic	America	18.9
36	https://www.cnn.com/2021/10/28/opinions/zuckerberg-facebook-meta-rushkoff/index.html	CNN	America	18.5
37	https://www.businessinsider.com/metaverse-zuckerberg-facebook-virtual-world-leave-people-behind-2021-12	Insider	America	18.1
38	https://www.bbc.com/news/technology-59154520	BBC	Britain	18
39	https://www.npr.org/2021/10/28/1049813246/facebook-new-name-meta-mark-zuckerberg	NPR	America	18
40	https://www.bbc.co.uk/news/technology-59154520	BBC	Britain	17.9
41	https://www.cbsnews.com/news/facebook-whistleblower-frances-haugen-metaverse-even-worse/	CBS News	America	17.5
42	https://www.insider.com/khaby-lame-touts-facebooks-metaverse-in-video-with-mark-zuckerberg-2021-11	Insider	America	17.4
43	https://venturebeat.com/2021/12/15/jadu-raises-7m-to-make-hoverboards-for-the-metaverse/	VentureBeat	America	17.2
44	https://www.coindesk.com/business/2021/10/28/facebooks-metaverse-will-support-nfts/	CoinDesk	America	17
45	https://mashable.com/article/iceland-roasts-mark-zuckerberg-meta-with-icelandverse	Mashable	America	16.3
46	https://www.coindesk.com/business/2021/10/12/jay-z-adds-incubator-to-portfolio-of-blockchaincrypto-investments-report/	CoinDesk	America	15.2
47	https://www.coindesk.com/business/2021/12/02/three-arrows-leads-43m-round-for-solana-based-metaverse-project-solice/	CoinDesk	America	15
48	https://www.theblaze.com/news/meta-metaverse-groping-facebook-investigation	Blaze Media	America	14.9
49	https://www.coindesk.com/business/2021/12/27/decentraland-luxury-marketplace-unxd-to-host-metaverse-fashion-week/	CoinDesk	America	14.5
50	https://www.cnbc.com/2021/12/15/millennials-gen-z-plan-to-spend-thousands-crypto-nfts-metaverse-land-as-holiday-gifts.html	CNBC	America	14.5
51	https://www.businessinsider.com/metaverse-facebook-hiring-10000-people-to-build-mark-zuckerbergs-project-2021-10	Insider	America	14.4
52	https://www.coindesk.com/business/2021/12/13/metaverse-company-infiniteworld-to-go-public-in-700m-spac-merger/	CoinDesk	America	14.2
53	https://venturebeat.com/2021/12/28/future-augmented-reality-will-inherit-the-earth/	VentureBeat	America	14.1
54	https://venturebeat.com/2021/08/21/jensen-huang-interview-the-physical-world-and-the-metaverse-can-be-connected/	VentureBeat	America	13.3
55	https://venturebeat.com/2021/12/26/how-ai-vr-ar-5g-and-blockchain-may-converge-to-power-the-metaverse/	VentureBeat	America	13.2
56	https://www.coindesk.com/business/2021/12/21/decentralized-rendering-engine-raises-30m-in-rndr-token-sale-as-metaverse-graphics-go-big/	CoinDesk	America	13
57	https://www.businessinsider.com/meta-woman-claims-virtually-groped-metaverse-horizon-venues-2022-1	Insider	America	12.1
58	https://apnews.com/article/facebook-meta-mark-zuckerberg-technology-business-5ad543ab7780caae435935f0aca9fac6	AP News	America	12
59	https://www.cnbc.com/2022/01/16/walmart-is-quietly-preparing-to-enter-the-metaverse.html	CNBC	America	11.8
60	https://www.zdnet.com/article/microsoft-to-extend-its-enterprise-metaverse-strategy-with-mesh-for-teams/	ZDNet	America	11.5
61	https://gizmodo.com/report-apple-wont-join-the-metaverse-hype-with-its-hea-1848331164	G/O Media - Gizmodo	America	11.3
62	https://www.ft.com/content/d3726a3a-c4a0-4761-a82c-cda98abc3cb7	Financial Times	Britain	10.7
63	https://thenextweb.com/news/apple-metaverse-off-limits-headset-analysis	The Next Web	Netherlands	10.5
64	https://www.coindesk.com/business/2021/12/22/dapper-coinfund-back-space-metaverse-play-with-7m-raise/	CoinDesk	America	10.5
65	https://www.ign.com/articles/playstation-creator-calls-vr-headsets-annoying-cant-see-the-point-metaverse	IGN	America	10.5
66	https://www.vice.com/en/article/wxdd74/zuckerbergs-metaverse-is-screwed-if-it-doesnt-allow-sex	Vice	America-Canada	10.3
67	https://venturebeat.com/2021/12/14/spatial-raises-25m-and-pivots-to-nft-art-and-metaverse-events/	VentureBeat	America	10.2
68	https://www.entrepreneur.com/article/393219	Entrepreneur	America	9.9
69	https://www.coindesk.com/business/2021/11/25/grayscale-says-metaverse-is-a-trillion-dollar-market-opportunity/	CoinDesk	America	9.9
70	https://futurism.com/the-byte/ar-pioneer-warns-metaverse	Futurism	America	9.9
71	https://venturebeat.com/2022/01/22/how-emerging-tech-will-influence-freedom-industry-and-money-in-the-metaverse/	VentureBeat	America	9.5
72	https://www.reuters.com/technology/facebooks-zuckerberg-kicks-off-its-virtual-reality-event-with-metaverse-vision-2021-10-28/	Reuters	Britain	9.5
73	https://www.breitbart.com/tech/2021/07/23/mark-zuckerberg-believes-facebook-will-usher-in-the-metaverse/	Breitbart	America	9.4
74	https://www.bbc.com/news/world-europe-58949867	BBC	Britain	9.4
75	https://www.coindesk.com/business/2022/01/28/fidelity-seeks-sec-approval-for-metaverse-etf/	CoinDesk	America	9.3
76	https://www.independent.co.uk/life-style/gadgets-and-tech/facebook-meta-name-new-metaverse-b1947245.html	Independent	Britain	9.3
77	https://www.bbc.co.uk/news/world-europe-58949867	BBC	Britain	9.1
78	https://www.theverge.com/22833369/nike-rtfkt-nft-sneaker-shoe-metaverse-company	The Verge	America	9.1
79	https://www.wsj.com/articles/mark-zuckerberg-to-sketch-out-facebooks-metaverse-vision-11635413402	The Wall Street Journal	America	8.5
80	https://www.coindesk.com/layer2/2021/12/21/web-3-and-the-metaverse-are-not-the-same/	CoinDesk	America	8.4
81	https://www.theguardian.com/technology/2021/oct/28/facebook-mark-zuckerberg-meta-metaverse	The Guardian	Britain	8.1
82	https://www.engadget.com/metaworld-nft-metaverse-dedric-reid-clubhouse-discord-170052007.html	Engadget	America	8.1
83	https://www.coindesk.com/markets/2021/12/15/hedera-a-distributed-network-targeting-businesses-jumps-into-metaverse-fray/	CoinDesk	America	8
84	https://www.reuters.com/technology/what-is-metaverse-2021-10-18/	Reuters	Britain	8
85	https://www.businessinsider.com/facebook-plans-rebrand-change-new-name-focus-metaverse-2021-10	Insider	America	8
86	https://www.rawstory.com/mark-zuckerberg/	Raw Story	America	8
87	https://futurism.com/metaverse-communist-propaganda	Futurism	America	7.7
88	https://www.bbc.com/news/technology-58749529	BBC	Britain	7.5
89	https://www.coindesk.com/business/2021/12/30/gamers-rally-behind-more-than-gamers-nft-project-and-its-metaverse-roadmap/	CoinDesk	America	7.4
90	https://www.reuters.com/business/media-telecom/us-reality-tv-star-paris-hilton-launches-metaverse-business-roblox-2021-12-28/	Reuters	Britain	7.4
91	https://www.cnbc.com/2021/12/31/shanghai-releases-five-year-plans-for-metaverse-development.html	CNBC	America	7.4
92	https://www.cnbc.com/2021/11/15/orlando-bravo-metaverse-is-investable-and-its-going-to-be-very-big.html	CNBC	America	7.2
93	https://www.bbc.co.uk/news/technology-58749529	BBC	Britain	7.2
94	https://futurism.com/facebook-concert-metaverse-flopped	Futurism	America	7
95	https://www.theguardian.com/commentisfree/2022/jan/29/the-metaverse-is-dystopian-but-to-big-tech-its-a-business-opportunity	The Guardian	Britain	6.9
96	https://www.reuters.com/technology/facebook-plans-hire-10000-eu-build-metaverse-2021-10-17/	Reuters	Britain	6.8
97	https://www.cnbc.com/2021/12/01/32-year-old-has-spent-6-figures-on-nfts-for-the-metaverse.html	CNBC	America	6.7
98	https://www.coindesk.com/business/2021/11/15/vcs-back-pawn-shop-of-the-metaverse-with-3m-raise/	CoinDesk	America	6.5
99	https://venturebeat.com/2021/11/17/mytaverse-launches-a-metaverse-for-business-travelers/	VentureBeat	America	6.4
100	https://www.businessinsider.com/meta-q4-earnings-largest-ever-one-day-wipeout-190-billion-2022-2	Insider	America	172.1
101	https://www.nytimes.com/2021/12/13/technology/instagram-handle-metaverse.html	New York Times	America	138
102	https://www.youtube.com/watch?v=enMwwQy_noI	Inspired by Iceland	Iceland	94.7
103	https://jacobinmag.com/2021/11/facebook-metaverse-mark-zuckerberg-play-to-earn-surveillance-tech-industry	Jacobin Magzine	America	93.8
104	http://www.autonews-mag.com/news/2025-emrehusmen-pickup-for-metaverse-future-electric-pickup	Auto Discoveries	America	83.8
105	https://www.cnbc.com/2021/10/28/facebook-changes-company-name-to-meta.html	CNBC	America	80.8
106	https://livesocial.deloitte.com/tL1I5k	The Verge	America	76.7
107	https://www.businessinsider.com/meta-metaverse-q4-zuckerberg-old-idea-never-worked-phil-libin-2022-2	Insider	America	65.5
108	https://autodiscoveries.com/2025-emrehusmen-pickup-for-metaverse-future-electric-pickup/	Auto Discoveries	America	65.4
109	https://www.youtube.com/watch?v=gElfIo6uw4g	CNET	America	64.7
110	https://www.cnbc.com/2022/02/02/meta-reality-labs-reports-10-billion-loss.html	CNBC	America	59
111	https://www.wired.co.uk/article/metaverse-big-tech-land-grab-hype	Wired	America	48.3
112	https://coinmarketcap.com/alexandria/article/facebook-metaverse-will-it-support-blockchain	CoinMarketCap	America	44.3
113	https://bigthink.com/the-future/metaverse-augmented-reality-danger/	Big Think	America	38
114	https://www.cnbc.com/2021/12/09/facebook-opens-horizon-worlds-vr-metaverse-app-.html	CNBC	America	37.6
115	http://cvsoci.al/qpu60NZO	CNBC	America	36.5
116	https://www.youtube.com/watch?v=Uvufun6xer8	Meta	America	36.2
117	https://deadline.com/2021/11/facebook-metaverse-meta-mark-zuckerberg-iceland-1234873459/	Deadline	America	34.7
118	https://www.forbes.com/sites/suzannerowankelleher/2021/11/12/video-icelandic-tourisms-epic-trolling-of-mark-zuckerberg-is-genuine-meta/	Forbes	America	33.6
119	https://www.technologyreview.com/2021/12/16/1042516/the-metaverse-has-a-groping-problem/	MIT Technology Review	America	29.8
120	https://www.breitbart.com/tech/2022/02/07/nyu-marketing-prof-blasts-mark-zuckerberg-facebooks-metaverse-is-a-giant-flaming-bag-of-sht/	Breitbart	America	29.3
121	https://www.youtube.com/watch?v=G8Cycty9Bg8	WSBDApp TV	America	26.8
122	https://futurism.com/the-byte/marketing-professor-metaverse	Futurism	America	25.9
123	https://finance.yahoo.com/news/cryptocity-metaverse-going-launch-3rd-172400288.html	Yahoo News	America	25.9
124	https://cybernews.com/news/disney-wants-to-become-the-happiest-place-in-the-metaverse/	Cyber News	Britain	24.3
125	https://www.vice.com/en/article/3abpg3/woman-says-she-was-virtually-gang-raped-in-facebooks-metaverse	Vice News	America	24.1
126	https://socialcentre.td.com/t4lKgt	Insider	America	23.3
127	https://www.businessinsider.com/facebook-meta-stock-price-market-colllapse-mark-zuckerberg-metaverse-mistake-2022-2	Insider	America	23.2
128	https://www.jacobinmag.com/2021/09/facebook-zuckerberg-metaverse-stephenson-big-tech	Jacobin Magzine	America	22.4
129	http://cvsoci.al/mylyUVso	CNBC	America	22.1
130	https://www.zdnet.com/article/facebooks-pivot-motivation-momentum-and-the-metaverse/	ZDNet	America	21.9
131	https://www.youtube.com/watch?v=ZJidR97VrcY	Triathon Lab	America	21.3
132	https://news.bitcoin.com/elon-musk-criticizes-current-state-of-web-3-wonders-about-future-of-metaverse/	Bitcoin.com	America	19
133	https://about.fb.com/news/2021/10/facebook-company-is-now-meta/	Meta	America	18.1
134	https://www.youtube.com/watch?v=n3WITHz4wvg	Devolution World Official	America	18
135	https://www.forbes.com/sites/michaeldelcastillo/2021/11/18/gemini-raises-400-million-to-protect-the-metaverse-from-meta-facebook/	Forbes	America	17.7
136	https://www.youtube.com/watch?v=Q_f83ANuN10	Radicle Gaming	America	17.4
137	https://english.alaraby.co.uk/news/metaverse-hajj-doesnt-count-turkish-authority-rules	The News Arab	Saudi Arabia	17.2
138	https://appleinsider.com/articles/21/12/31/interest-in-the-metaverse-to-propel-apple-meta-in-2022-analyst-says	Apple	America	17
139	https://babylonbee.com/news/god-develops-ultra-realistic-metaverse-where-people-can-talk-learn-and-work-with-other-people-calling-it-universe	The Babylon Bee	America	16.7
140	https://www.youtube.com/watch?v=g3m22SF-9WM	Phantasma Chain	Portugal	15.4
141	https://afrotech.com/khaby-lame-facebook-metaverse	AFRO Tech	America	14.9
142	https://www.dailywire.com/news/meta-facebooks-new-name-mocked-online-means-dead-in-hebrew	The Daily Wire	America	115.6
143	https://www.vice.com/en/article/88g9vv/zuckerbergs-meta-endgame-is-monetizing-all-human-behavior	Vice	America	57.4
144	https://www.theverge.com/2021/10/29/22753046/facebook-meta-aoc-instagram-rebrand-france-haugen-blumenthal	The Verge	America	57.3
145	https://www.thedailybeast.com/in-latest-indignity-at-meta-one-time-facebook-employees-will-now-be-called-metamates	The Daily Beast	America	40.8
146	https://www.businessinsider.com/meta-facebook-user-numbers-shrink-first-time-ever-2022-2	Insider	America	34.9
147	https://www.reuters.com/technology/facebook-owner-meta-forecasts-q1-revenue-below-estimates-2022-02-02/	Reuters	Britain	33.6
148	https://www.nytimes.com/2022/02/03/technology/facebook-meta-challenges.html	New York Times	America	29.6
149	https://venturebeat.com/2021/08/24/alethea-ai-raises-16m-in-private-token-sale-for-intelligent-nfts-for-the-metaverse/	VentureBeat	America	29.4
150	https://www.businessinsider.com/facebook-papers-inside-meta-years-of-scandals-leaked-documents-2021-11	Insider	America	29
151	https://www.indiatoday.in/technology/news/story/woman-complains-she-was-virtually-groped-on-meta-s-horizon-worlds-company-says-improving-interface-1889975-2021-12-20	India Today	India	28
152	https://globalnews.ca/news/8332537/facebook-mark-zuckerberg-metaverse-conference/	Global News	Canada	27
153	https://www.businessinsider.com/facebook-says-apple-sony-microsoft-google-are-metaverse-competition-2021-11	Insider	America	25.3
154	https://www.businessinsider.com/facebook-meta-rebrand-damaged-reputation-study-finds-2021-11	Insider	America	25.2
155	https://www.newstatesman.com/the-business-interview/2021/11/we-must-prevent-facebook-from-running-the-metaverse-herman-narula-on-the-fight-for-a-new-reality	The News Statesman	Britain	24.5
156	https://www.engadget.com/horizon-worlds-now-available-us-canada-192520479.html	Engadget	America	24.1
157	https://www.coindesk.com/business/2022/02/16/snoop-dogg-plans-to-turn-death-row-records-into-the-first-nft-music-label/	CoinDesk	America	23
158	https://futurism.com/the-byte/people-entire-lives-metaverse	Futurism	America	22.3
159	https://hbr.org/2022/01/how-brands-can-enter-the-metaverse	Harvard Business Review	America	22
160	https://www.zdnet.com/article/facebook-brings-metaverse-to-work-with-horizon-workrooms-and-you-thought-zoom-fatigue-was-bad/	ZDNet	America	21
161	https://arstechnica.com/gaming/2022/02/meta-establishes-four-foot-personal-boundary-to-deter-vr-groping/	Ars Technica	America	20.9
162	https://www.reuters.com/technology/metaverse-pioneers-unimpressed-by-facebook-rebrand-2021-11-01/	Reuters	Britain	20.9
163	https://www.cnbc.com/2022/01/13/analysts-on-metaverse-benefiting-chipmakers-big-tech-crypto.html	CNBC	America	20.2
164	https://www.coindesk.com/business/2022/02/15/jpmorgan-is-the-first-bank-into-the-metaverse-looks-at-business-opportunities/	CoinDesk	America	19.5
165	https://www.cnbc.com/2022/02/01/metaverse-real-estate-sales-top-500-million-metametric-solutions-says.html	CNBC	America	18.1
166	https://venturebeat.com/2021/04/13/epic-games-raises-1-billlion-to-fund-long-term-metaverse-plans/	VentureBeat	America	18.1
167	https://www.cnbc.com/2021/12/22/here-are-the-companies-building-the-metaverse-meta-roblox-epic.html	CNBC	America	17.3
168	https://www.coindesk.com/business/2021/11/22/metaverse-gaming-nfts-could-account-for-10-of-luxury-market-by-2030-morgan-stanley/	CoinDesk	America	17
169	https://www.coindesk.com/layer2/2021/12/14/get-ready-for-the-2022-metaverse-real-estate-boom/	CoinDesk	America	17
170	https://gizmodo.com/iceland-skewers-mark-zuckerbergs-metaverse-with-new-tou-1848044804	G/O Media - Gizmodo	America	17
171	https://www.reuters.com/technology/facebook-launches-vr-remote-work-app-calling-it-step-metaverse-2021-08-19/	Reuters	Britain	16.9
172	https://www.businessinsider.com/facebook-metaverse-meta-zuckerberg-physical-store-brick-mortar-2021-11	Insider	America	16.9
173	https://www.vox.com/recode/22799665/facebook-metaverse-meta-zuckerberg-oculus-vr-ar	VOX	America	16.7
174	https://www.theguardian.com/technology/2022/feb/04/meta-to-bring-in-mandatory-distances-between-virtual-reality-avatars	The Guardian	Britain	16.7
175	https://www.pcmag.com/opinions/the-real-5g-metaverse-will-blow-facebooks-meta-out-of-the-water	PC Magzine	America	16.5
176	https://www.coindesk.com/business/2021/11/18/time-magazine-to-hold-eth-on-balance-sheet-as-part-of-galaxy-digital-metaverse-deal/	CoinDesk	America	16.4
177	https://www.theverge.com/22749919/mark-zuckerberg-facebook-meta-company-rebrand	The Verge	America	16.3
178	https://www.newsweek.com/mother-alleged-she-was-virtually-groped-attack-inside-facebooks-metaverse-1674718	News Week	America	16.3
179	https://www.cnbc.com/2021/11/02/nike-is-quietly-preparing-for-the-metaverse-.html	CNBC	America	16.1
180	https://www.engadget.com/apple-no-metaverse-for-vr-headset-182544991.html	Engadget	America	16
181	https://www.coindesk.com/business/2021/12/17/goldman-sachs-says-blockchain-is-key-to-metaverse-and-web-3-development/	CoinDesk	America	16
182	https://www.ign.com/articles/facebook-rebrand-its-name	IGN	America	16
183	https://www.theatlantic.com/technology/archive/2021/10/facebook-metaverse-was-always-terrible/620546/	The Atlantic	America	16
184	https://www.cnbc.com/2021/11/05/meta-is-reportedly-planning-retail-stores-as-it-pushes-into-metaverse.html	CNBC	America	15.9
185	https://www.theverge.com/2021/11/2/22758974/microsoft-teams-metaverse-mesh-3d-avatars-meetings-features	The Verge	America	15.9
186	https://www.vox.com/recode/22933851/meta-facebook-metaverse-antitrust-regulation	VOX	America	15.9
187	https://arstechnica.com/gaming/2021/10/john-carmack-sounds-a-skeptical-note-over-metas-metaverse-plans/	Ars Technica	America	15.7
188	https://www.dailywire.com/news/facebook-announces-50-million-investment-promises-to-build-metaverse-responsibly	The Daily Wire	America	15.7
189	https://www.washingtonpost.com/technology/2021/08/30/what-is-the-metaverse/	Washington Post	America	15.6
190	https://www.bloomberg.com/news/newsletters/2022-02-11/the-metaverse-makes-no-sense-and-here-s-why	Bloomberg	America	15.5
191	https://www.theguardian.com/technology/2022/feb/15/meta-mark-zuckerberg-facebook-metaverse	The Guardian	Britain	15.5
192	https://www.bbc.com/news/technology-57942909	BBC	Britain	15.4
193	https://markets.businessinsider.com/news/currencies/metaverse-property-decentraland-sale-price-mana-coin-cryptocurrency-2021-11	Insider	America	15.2
194	https://www.theguardian.com/commentisfree/2021/oct/29/facebook-reality-metaverse-mark-zuckerberg	The Guardian	Britain	15.2
195	https://www.theblaze.com/news/facebook-company-name-changed-to-meta-as-zuckerberg-plans-to-focus-on-developing-the-metaverse	Blaze Media	America	15.2
196	https://www.businessinsider.com/zuckerberg-said-facebook-will-eventually-become-a-metaverse-company-2021-7	Insider	America	15.2
197	https://www.businessinsider.com/facebook-metaverse-frances-haugen-whistleblower-shocked-by-investment-2021-10	Insider	America	15
198	https://www.businessinsider.com/meta-metaverse-virtual-groping-personal-boundary-safety-bubble-horizons-venues-2022-2	Insider	America	15
199	https://www.reuters.com/investigates/special-report/finance-crypto-sundaresan/	Reuters	Britain	15
200	https://www.reuters.com/business/metaverse-bet-crypto-rich-investors-snap-up-virtual-real-estate-2021-04-19/	Reuters	Britain	15
201	https://www.wired.com/story/metaverse-land-rush-illusion/	Wired	America	14.9
202	https://www.rollingstone.com/music/music-news/foo-fighters-perform-vr-set-1296186/	Rolling Stone	America	14

## Appendix B codebook instructions

Code each assigned online news according to the following instructions:

A. Frame element one: problem definition

Description: This element determines the costs and benefits of the metaverse mentioned in the news, usually measured in terms of common cultural values.

Code 1: Benefits - consumer
  Description: Metaverse is portrayed as a benefit for consumers, including but not limited to remote work, study, shop, travel; immersive internet experience, alternative lifestyle, etc.
  Example 1: In one news titled “Bill Gates says most virtual meetings will move to the metaverse within 3 years, and workers will interact using VR headsets and avatars”, it described the benefit of metaverse for a more interactive remote work meeting.
  Example 2: In one news titled “Facebook CEO Zuckerberg renames company Meta, outlines vision for Horizon' metaverse”, it mentioned the benefit of Facebook's metaverse for opening up new possibilities of e-learning, the exact words were “Horizon will also be used for educational purposes, allowing teachers and parents to transport their kids to different eras and more.”
Code 2: Benefits - economics
  Description: Metaverse is referred to as a benefit for economics, including but not limited to full-functioning economies online, opportunities to new kinds of business for either existing companies or new emerging companies, creating more job opportunities, etc.
  Example 1: In the news titled “Metaverse Startup The Sandbox Closes $93M Series B Led by SoftBank”, it proposed one benefit of the metaverse – opportunities for new business, like the startup company the Sandbox.
  Example 2: In the news titled “Microsoft to goggle up Activision in $69 billion metaverse bet”, it points out many big companies are also building their metaverse and joined the business competition.
  Example 3: In the news titled “Metaverse real estate sales top $500 million, and are projected to double this year”, it indicated the economical potential of the metaverse.
Code 3: Risks - ethics
  Description: Metaverse is regarded as a risk for ethics, including but not limited to privacy, data concerns, misinformation/disinformation, divisiveness, polarization, autonomy, etc.
  Example 1: The news titled “The metaverse could let Silicon Valley track your facial expressions, blood pressure, and your breathing rates — showing exactly why our internet laws need updating” expressed the concern over users' data in the metaverse.
  Example 2: The news titled “Metaverse, Mars, meditation retreats: billionaires want to escape the world they ruined” criticized the unethical conduct of Zuckerberg's metaverse as a way to sway audiences' attention from the damages they have already caused to the society in the real world and uttered the worry of unethical behaviours of Facebook in the metaverse as well.
  Example 3: The news titled “Facebook whistleblower fears Meta's plan for the metaverse” pointed out the unethical deeds of Zuckerberg's metaverse that they prioritize profits over users' safety and the worry of more serious situation in the metaverse, like misinformation and disinformation.
Code 4: Risks - wellbeing
  Description: Metaverse is treated as a risk for wellbeing, including but not limited to online addiction, distorted sense of time and space, dysfunctional interpersonal relationship, etc.
  Example 1: The news titled “Former Google CEO says Facebook's metaverse is 'not necessarily the best thing for human society' and expresses concerns about safety of artificial intelligence” stated that “Schmidt said he views AI, which Meta uses to run a majority of its platforms' algorithms, as a "giant, false god" that can create unhealthy and parasocial relationships.”
  Example 2: The news titled “A woman claimed she was virtually groped by a gang of male avatars in Meta's metaverse, report says” gave an example of women's wellbeing risk in the metaverse.
Code 5: Risks - out of control
  Description: Metaverse is described as a risk to go out of control, and take over humanity, etc.
  Example 1: The news titled “What Zuckerberg's metaverse means to our humanity” mentioned that “No, to get through the portal to Facebook's metaverse, to go in the direction that Zuckerberg is pushing us, we must leave our humanity behind.” It articulated the worry of human being replaced by algorithms, robotic, and technology.
  Example 2: The news titled “Singularity Is Fast Approaching, and It Will Happen First in the Metaverse” predicted and warned that singularity will happen in the metaverse where “artificial intelligence supersedes human intelligence and will eventually escape our control. Some even predict catastrophic consequences for humanity where machines will become the dominant species on this planet.”
Code 6: Challenges
  Description: Metaverse is framed as a challenging futuristic idea to achieve, including but not limited to technical challenges, public acceptance challenges, etc.
  Example 1: The news titled “Chinese tech giant Baidu says it could be 6 years before it can fully deliver its metaverse” expressed that the metaverse is still in its infancy and that it needs more time than expected to fully deliver that: “However, at a preview event Tuesday, the executive in charge of XiRang downplayed expectations by noting how many aspects weren't yet up to par.”
  Example 2: In the news titled “NYU Marketing Prof Blasts Mark Zuckerberg: Facebook's Metaverse Is a ‘Giant Flaming Bag of Sh*t’”, Galloway stated that one of the biggest issues for Zuckerberg is that the company's Quest VR headset is still too clunky and awkward to convince users to adapt to the metaverse.
B. Frame element two: causal diagnosis
  Description: This frame element identifies the forces creating the problem, either benefits, risks, or challenges.
Code 7: Benefits - technology
  Description: Metaverse itself is responsible for the benefit.
  Example 1: The news titled “Bill Gates says the metaverse will host most of your office meetings within ‘two or three years’ — here's what it will look like” complimented the metaverse role in solving remote work problems especially during this pandemic and attributed this benefit to the metaverse: “In a year-end post on Gates' personal blog, the Microsoft co-founder included a prediction about the future of work, and how it could be changed by the rise of the metaverse.”
  Example 2: The news titled ““Metaverse real estate sales top $500 million, and are projected to double this year” outlined the economical benefit of the metaverse for new market and attributed this to the metaverse.
Code 8: Benefits - business
  Description: Business actors, such as tech companies and industry, are responsible for the benefit.
  Example 1: The news titled "Facebook to hire 10,000 in EU to work on metaverse” attributed the new job opportunities to Facebook that is working on the metaverse.
  Example 2: The news titled “Facebook CEO Zuckerberg renames company Meta, outlines vision for Horizon' metaverse” attributed the benefit of the metaverse for e-learning/work to Facebook's investment into building such a place.
Code 9: Risks - technology
  Description: Metaverse itself is responsible for the risk.
  Example 1: The news titled “Singularity Is Fast Approaching, and It Will Happen First in the Metaverse” raised concern for singularity where “the exponential acceleration of technological development will lead to a situation where artificial intelligence supersedes human intelligence and will eventually escape our control” and attributed this risk to the technology.
  Example 2: The news titled “The Metaverse Needs a Constitution” expressed concern over the risks of data concern, misinformation, etc. and attributed that to the metaverse: “Emerging technologies have a way of solving old problems whilst creating new ones.”
Code 10: Risks - business
  Description: Business actors, such as tech companies and industry, are responsible for the risk.
  Example 1: The news titled “A woman claimed she was virtually groped by a gang of male avatars in Meta's metaverse, report says” gave an example of women's wellbeing risk in the metaverse and asked for meta's responses and reactions to this risk. It attributed this safety problem to the company.
  Example 2: The news titled “What Zuckerberg's metaverse means to our humanity” raised concern over humanity problem and attributed it to Zuckerberg's wrong direction and action into the metaverse.
Code 11: Challenges - technology
  Description: Metaverse itself is responsible for the challenge.
  Example 1: The news titled “Chinese tech giant Baidu says it could be 6 years before it can fully deliver its metaverse” acknowledged that the metaverse requirement itself caused the technical challenges to build the metaverse.
  Example 2: The news titled “Apparently, it's the next big thing. What is the metaverse?” pointed out that the metaverse was challenging to achieve and still need long way to evolve. And it attributed this challenge to technology itself because of its high requirements, not any actors.
Code 12: Challenges - business
  Description: Business actors, such as tech companies and industry, are responsible for the challenge.
  Example 1: The news titled “NYU Marketing Prof Blasts Mark Zuckerberg: Facebook's Metaverse Is a ‘Giant Flaming Bag of Sh*t’” pointed out that the reason why people don't like or accept Facebook's metaverse was that “Galloway doesn't believe that the company is going in the right direction.” The company was attributed for its challenges to gain public acceptance.
  Example 2: In the news titled “Report: Apple Won't Join the Metaverse Hype With Its Headset”, it said that “It sounds like Apple CEO Tim Cook won't follow the lead of Facebook, er, Meta, and just about every other company that has chimed in recently to discuss the ways they can bring us closer to a poorly defined, ill-conceived future where the physical and digital world melt together via the use of VR, AR, and mixed reality.” It attributed the metaverse problem to the business company leading the metaverse who just created the concept as a buzzword/hype but poorly defined and ill-conceived.
C. Frame element three: moral evaluation
  Description: This frame element evaluates metaverse and its effects in the news.
Code 13: desirable
  Description: The main tone of the news is pro-metaverse and emphasizes on its promising economic and social prospects.
  Example 1: The news titled “Epic Games raises $1B for long-term metaverse plans, with $200M from Sony” described the fund raise of the company for the metaverse plan. It set the major tone of the metaverse as a desirable chance that many companies are joining the competition.
  Example 2: The news titled “Metaverse real estate sales top $500 million, and are projected to double this year” focused on the large market of the metaverse and portrayed it as a desirable opportunity for the business.
Code 14: threatening
  Description: The main tone of the news is against-metaverse and emphasizes on its threats on people, society, and humanity.
  Example 1: The news titled “Metaverse: Augmented reality pioneer warns it could be far worse than social media” highlighted the threatening consequences of the metaverse to people, society, and humanity.
  Example 2: The news titled “Mark Zuckerberg's metaverse could fracture the world as we know it — letting people 'reality block' things they disagree with and making polarization even worse” emphasized the threatening effect of the metaverse on polarization and disappearing reality.
Code 15: unwanted
  Description: The main tone of the news is against-metaverse and highlights people's unwillingness and low interest in the metaverse because of its drawbacks/shortcomings/flaws.
  Example 1: The news titled “The inventor of PlayStation thinks the metaverse is pointless” set the tone of the metaverse as useless and meaningless thing to people.
  Example 2: The news titled “Mark Zuckerberg is creating a future that looks like a worse version of the world we already have” had a major tone of against the metaverse and low interest in the metaverse.
  Example 3: The news titled “NYU Marketing Prof Blasts Mark Zuckerberg: Facebook's Metaverse Is a ‘Giant Flaming Bag of Sh*t’” focused the shortcomings of the metaverse and pointed out that people don't want it.
Code 16: probable
  Description: There is no incline in pro or against the metaverse. Both sides of the story are displayed in the news.
  Example 1: The news titled “The Metaverse Needs a Constitution” expressed that the metaverse may have certain problems but if addressing them properly, the metaverse could still work. It did not simply come to a conclusion on yes or no to the metaverse.
  Example 2: The news titled “Facebook is rebranding as Meta — but the app you use will still be called Facebook” merely stated the fact of Facebook's rebranding and analysed this event from both sides without leading audiences to go either pro or against the metaverse.
D. Frame element four: treatment recommendation
  Description: This frame element offers and justifies treatments for the problems of the metaverse.
Code 17: investment
  Description: The news calls for further investment to achieve metaverse, like money, attention, time, thoughts, and various resources to help build a metaverse.
  Example 1: The news titled “Enjin Forms $100M Fund to Support Metaverse Projects” implied that there existed many opportunities in the metaverse and you should come aboard and invest in it.
  Example 2: The news titled “Millennials and Gen Z are planning to spend thousands on cryptocurrency, NFTs and metaverse land as holiday gifts” indicated that there was a huge market in the metaverse and it worth your investment.
Code 18: regulation
  Description: The news brings up the need for laws, regulations, standards, guidelines, principles, and all other kinds of written or socially agreed rules for the metaverse.
  Example 1: The news titled “The Metaverse Needs a Constitution” uttered a need of a constitution to regulate the metaverse.
  Example 2: The news titled “The metaverse could let Silicon Valley track your facial expressions, blood pressure, and your breathing rates — showing exactly why our internet laws need updating” requested rules and regulations on unethical data collection from the tech companies.
Code 19: stop
  Description: The news proposes to stop any kinds of behaviours in propelling the metaverse.
  Example 1: The news titled “Facebook's “Metaverse” Must Be Stopped” directly proposed to stop the metaverse.
  Example 2: The news titled “The inventor of PlayStation thinks the metaverse is pointless” pointed out that the metaverse is not welcomed by the public and did nothing good to people. It implied that the metaverse is not a good investment and should stop.
Code 20: debate
  Description: The news doesn't offer any treatment itself. It just offers fair information of two sides of the metaverse or its related actions without inclining to any side in order to foster public debate.
  Example 1: The news titled “Facebook is rebranding as Meta — but the app you use will still be called Facebook” didn't give any suggestions for the problem. It just gave people information and raised people to think about the consequences and draw their conclusions.
  Example 2: The news titled “MARK IN THE METAVERSE: Facebook's CEO on why the social network is becoming ‘a metaverse company’” guided people to think deeper on the motives of Facebook's metaverse. It didn't propose any treatment directly or implicitly.

## Footnotes

1 Retrieved on 15.01.2022 at https://trends.google.com/trends/explore?date=all&q=metaverse.
